# Tell Me about Loneliness: Interviews with Young People about What Loneliness Is and How to Cope with It

**DOI:** 10.3390/ijerph182211904

**Published:** 2021-11-12

**Authors:** Lily Verity, Tine Schellekens, Tine Adam, Floor Sillis, Marinella Majorano, Michael Wigelsworth, Pamela Qualter, Beth Peters, Stephanie Stajniak, Marlies Maes

**Affiliations:** 1 School of Environment, Education and Development, The University of Manchester, Manchester M13 9PL, UK; michael.wigelsworth@manchester.ac.uk (M.W.); pamela.qualter@manchester.ac.uk (P.Q.); beth.peters@student.manchester.ac.uk (B.P.); stephanie.stajniak@student.manchester.ac.uk (S.S.); 2 School Psychology and Development in Context, KU Leuven, Oude Markt 13, 3000 Leuven, Belgium; tine.schellekens@kuleuven.be (T.S.); tine.adam@student.kuleuven.be (T.A.); floor.sillis@student.kuleuven.be (F.S.); marlies.maes@kuleuven.be (M.M.); 3 Department of Human Sciences, University of Verona, Francesco, 37129 Verona, Italy; marinella.majorano@univr.it

**Keywords:** adolescence, loneliness, qualitative

## Abstract

Background: loneliness is a common experience for adolescents, yet the voices of adolescents are missing from current conceptualisations of loneliness. That means, measures that have been created based on current conceptualisations may miss important contexts of adolescence, such as the roles of friendships, that determine the way loneliness is experienced. The current study aims to centre adolescent voices to identify how they conceptualise loneliness and what strategies they consider to be useful for adolescents to cope with loneliness. Method: thematic framework analysis (TFA) was conducted on qualitative interviews with young people aged 8–14 years in Belgium and Italy to identify salient themes in their conceptualisations of loneliness. Results: Loneliness was conceptualised as a negative emotional state involving negative thinking patterns that occurs when an individual perceives they are missing out on a desired aspect in their social relationships. Coping strategies related to alleviating negative affect, and aiding social reconnection. Conclusions: friendships with peers were understood to be central to adolescent loneliness experiences. In line with that, loneliness was seen to be experienced at school. Age-related differences in friendship expectations were identified, highlighting how developmental needs relate to the loneliness experience.

## 1. Introduction

The social contexts of adolescence and adulthood are very different, and it is likely loneliness is conceptualised dissimilarly according to the age at which it is experienced [1]. However, the most frequently used measures of loneliness for adolescents have been informed only by research investigating the experiences and conceptualisations of loneliness in adulthood, and missing from the development of those measures are the voices of adolescents [2]. That has implications for current interventions for adolescents who report loneliness because they neglect to consider how interventional strategies that are beneficial in adulthood may be incongruent within the contexts of adolescence [3]. The current study “returns to basics”: it uses qualitative methods to investigate young people’s experiences and understanding of loneliness and how they cope with loneliness by asking them directly. Consequently, we provide a unique contribution to understanding loneliness among youth that can inform further research in the development of loneliness measures and intervention materials that are tailored towards young people’s experiences of loneliness, which contrasts with current practice, where ideas developed from older adults are applied to a younger population.

Loneliness is an issue prevalent and common across the life course, peaking in both adolescence and older adulthood [4]. It has been defined as a negative emotional experience that occurs when someone perceives a discrepancy between their actual social relationships, and those they desire; that discrepancy can relate to quality or quantity of relationships [5]. Loneliness is often wrongly conflated with social isolation; research identifies that loneliness can occur in the presence of others, even others who we have social connections with, and spending time alone is not guaranteed to result in loneliness [6]. It is also distinct from solitude, whereby a person desires alone time: loneliness is not considered to be a positive experience [7]. Loneliness is theorised to be an evolutionary mechanism that alerts individuals to seek connection or reconnection with others; that enhances safety by ensuring belongingness to a group, as opposed to being alone and therefore vulnerable to threat [8]. However, for some, loneliness can become chronic, and leave them stuck in a loop of hypervigilance to social threat, maladaptive thinking, and strained connections with others [6]. A recent meta-synthesis of experiences of loneliness among young people with depression highlighted a paradox of yearning to be with others, whilst wanting to withdraw and be alone [9].

Weiss developed the social and emotional loneliness typology that posits that loneliness can be broken down into: (1) social loneliness, where an individual feels lonely due to a lack of a social network, and (2) emotional loneliness, where an individual feels lonely due to a lack of quality intimate relationships [10]. Those conceptualisations were developed based on knowledge of loneliness experiences in older adulthood [11]. Because loneliness is prevalent in young people, and the contexts of youth differ to those of older adulthood, it is important to establish whether such conceptualisations can be applied to experiences of loneliness in young people by centring their voices in research [12].

The function of social relationships changes throughout the lifespan, with attachments to caregivers being more salient for young people than older adults [13]. The role of friendships also changes throughout the lifespan, moving from playmates to confidants as young people move through childhood to adolescence [14]. Creating friendships is a focus of adolescence, but older adults narrow their social networks, benefiting from fewer emotionally close partnerships [15]. Nicolaisen and Thorsen highlight that young adults have higher expectations for frequency of contact, companionship, and intimacy from friends than older adults, and even those who see their friends regularly are often disappointed by their interactions [16]. It can be assumed then, that even those young people who are in regular contact with friends are at risk of experiencing loneliness. Despite that, interventions for loneliness in young people have tended to target individuals with social skills deficits based on the misguided assumption that youth loneliness is a result of not being able to make friends [17].

Friendship in youth is not a homogenous experience: it is influenced by individual differences such as gender [18]. Expectations of friendships in adolescence differ according to gender; girls express a greater need for communal friendship, and receive greater communal friendship supports than boys [18]. Girls are more likely than boys to talk about their problems, and more likely to perceive that talk positively [19]. In addition, social withdrawal is received more negatively by peers if enacted by a male than a female [20]. We might expect then that the greater expectations for female friendships mean that female youth are more likely to experience loneliness, but that conversely, support in coping with loneliness may be more readily available to females than males.

Understanding how young people experience loneliness can help to enhance the validity of measurements for loneliness and to identify strategies that young people consider useful for overcoming loneliness that can be utilised in interventions [21]. Quantitative research has provided insights into the factors associated with loneliness in youth, but what is missing is how youth understand loneliness. Examining the meaning that young people give to loneliness can help to establish the appropriate utility of the concept in psychological research, i.e., whether measurements of loneliness, particularly social and emotional loneliness, are appropriate for use with young people. The current study builds on previous qualitative research with young people (8- to 18-year-olds) to understand conceptualisations of loneliness among young people. Previous research that used thematic framework analysis (TFA) [22] to analyze online counselling conversations with young people experiencing loneliness identified that young people experience loneliness as a negative, emotive experience that is “dark” and “haunting” [23]. Furthermore, young people experiencing loneliness struggled to know how to successfully cope with it, but the authors were unable to ask direct questions that could provide more insight about young peoples’ thoughts about loneliness due to the nature of the data collected on-line during chats with counsellors. The current study asks youth directly how they would cope with loneliness. Although the youths in the current study were not always experiencing loneliness, it can be assumed that the suggestions they make would be the strategies they would undertake, if they were to feel lonely.

Previous qualitative research interviewed youths about their conceptualisations of loneliness and concluded that it was a multifactorial experience that focused on connectedness with friends and perceptions of aloneness [24]. Individual perceptions of their social state were also important in previous qualitative research, which examined experiences of loneliness in adolescent “outsiders”. That work highlighted: (1) that those who identified themselves as being an “outsider” considered this to be their own choice, but they otherwise could have been misidentified as lonely from an exterior perspective [25], and (2) the importance of focusing on aspects of loneliness beyond solely social isolation, to identify how loneliness differs to social isolation for youth. The current study expands on that previous work by exploring individual perspectives on loneliness in more depth. TFA was used to identify themes that provide a wider reaching narrative about the data. The study aimed to (1) establish how young people conceptualise loneliness, (2) explore whether they consider there to be any gender differences in experiences of loneliness, and (3) how they suggest that young people, and those around young people can cope with loneliness.

## 2. Materials and Methods

Semi-structured interviews were conducted with 12 (female = 6, male = 6) young people aged 8–14 years at a school in Belgium, and with 12 (female = 6, male = 6) young people aged 9–12 years at a school in Italy. The age range corresponds with age limits utilised in research on middle childhood and adolescence: 6–18 years [26]. The study used a stratified sample: for each age group, participants were randomly selected from their school year group for the interview, split equally by gender. All 24 interviews were included in the analysis. For the Belgian data collection, interviews were conducted face to face; the participating primary school is located in the province of East Flanders: the participating secondary school in the province of Antwerp. All participants spoke fluent Dutch. No further information was obtained about the ethnic background of the participants. The Italian participants were recruited from one school in the North of Italy. Ethical approval was obtained from KU Leuven, and the University of Verona for the Belgian and Italian data collection, respectively.

### 2.1. Materials

The team devised an interview protocol for the semi-structured interviews. Those were adapted to the age categories they targeted resulting in two separate protocols for children and adolescents (see Appendix A). The duration of each interview was between 20–40 min.

### 2.2. Data Collection

Two team members were responsible for conducting the Belgian interviews, and one other team member conducted the Italian interviews. Ten Belgian interviews were conducted face to face before COVID-19 social restrictions were in place; the Italian data were collected during the COVID-19 lockdown in Italy via Zoom online meetings; two further Belgian interviews were conducted face to face after approximately 1 year of COVID-19 social restrictions in Belgium.

### 2.3. Data Analysis

Prior to analysis, interviews were transcribed, then translated from Dutch and Italian to English. TFA (Further details on the analytical process can be found in Appendix B) was conducted to analyze the data for themes that reflected how young people talked about what they thought loneliness was, how they thought someone with loneliness might feel, and what they thought could be done to help someone overcome their feelings of loneliness. First, the Belgian team read through each of their transcripts (in Dutch) and summarised those and identified potential initial codes, while the United Kingdom (UK) team did the same for both the Belgian and Italian transcripts (in English). Both teams then came together to discuss those summaries and potential initial codes. The UK team organised those summaries and codes into an initial chart of data in Microsoft Excel in order for the whole team to look more thoroughly at potential codes and develop a list of initial codes. The UK team and Belgian team each created a list of initial codes that were then consolidated into a coding framework that organised codes into categories and included definitions. That framework was then trialed on one Belgian transcript, and one Italian transcript. Group meetings were held to discuss those trials to determine how to unify our approach to coding, and further develop the coding framework. That process was repeated three times until we were satisfied that the framework reflected the data set as a whole. We then applied the finalised coding framework to the whole data set; Belgium coded their transcripts in Dutch, and the UK coded the Italian transcripts in English. Both teams transferred the data into a Microsoft Excel file where all the coded data were translated into English in order for the whole team to be able to interpret the data and develop themes. The UK and Belgian teams created lists of emerging themes that were then discussed as a whole team and refined, with quotes chosen to support the final themes. Whilst the consensus-based approach to analysis might appear to limit generalizability, the aim of the process was to enhance the applicability of findings, rather than generalizability. The analytical approach included continual reappraisal of the data by the team in order to ensure that interpretations were representative of the data set as a whole, as opposed to focusing overly on transcripts that may reiterate researchers’ pre-existing biases about loneliness. The transparency enabled by TFA means that judgements are made by those assessing the applicability of findings to their setting [27].

Following the TFA, we conducted secondary analyses to examine gender differences in participants’ suggestions for ways to cope with loneliness, and age differences in conceptualisations of the experience of loneliness. Secondary analyses consisted of deductive content analysis (Further details on the analytical process can be found in Appendix B) devised from findings produced by the TFA.

## 3. Results

**Figure 1:**
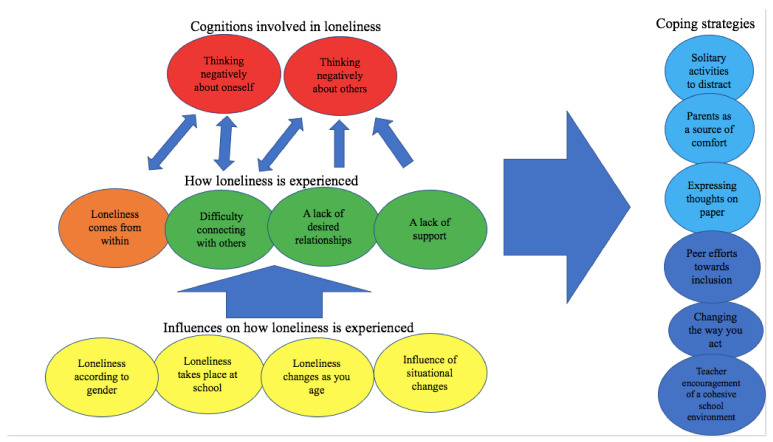
Establishing connections between themes. The figure shows a concept map which illustrates how interpretations are linked together. The arrows show the directions in which the concepts impact upon each other; the different colors represent the themes to which the subthemes depicted belong.

Using TFA, we identified six themes (and 12 subthemes) that illustrate the ways young people described loneliness and suggestions they make for how to cope with it: 1. Loneliness Comes from Within, 2. Loneliness as Missing Out, 3. Loneliness and Thinking Negatively, 4. Loneliness as a Transient Experience, 5. Coping Strategies That Aim to Alleviate the Negative Emotional Impact of Loneliness, and 6. Coping Strategies That Aid Social Reconnection. An illustration of how the themes developed are connected can be seen in Figure 1.

### 3.1. Loneliness Comes from Within

Participants described loneliness as a negative emotional state that involved feelings of sadness. Initially, some participants faced difficulties in differentiating how loneliness was different to being alone:

“that you, that that is someone who is alone, that he has no one around him. [hmhm] And that [pause] is separated from the rest or something” (Belgian, male, 14 years).

This indicated that some participants had potentially not thought about loneliness in depth before. After more discussion, they identified that loneliness could occur even when in the presence of others, but that loneliness often occurred because someone felt, or was excluded from social situations:

“When people feel lonely, they are usually sad because they feel excluded [because no one wants to play with them]” (Belgian, Female, 10 years).

“Maybe when the group of friends doesn’t accept you for something, like that, or when there are no friends around to help you” (Belgian, male, 9 years).

Although participants recognised that loneliness often occurred alongside being alone, Italian participants, in particular, described loneliness in regard to internal thoughts and feelings, as opposed to being determined by physical isolation. That meant, Italian participants identified that loneliness could occur if a person felt negatively about their situation, regardless of whether they had positive relationships with those around them.

“well feeling lonely can be… you think that nobody wants you for some reason, but instead there are a lot of people who want you, even if you tend to be alone” (Italian, male, 12 years).

“being lonely is something you feel, but maybe...and…you’re not and instead being alone is that you’re alone and you have no one” (Italian, male, 9 years).

“to feel lonely is anyway…that you have someone though...you think I don’t help you, but instead it’s not true like...” (Italian, female, 12 years).

### 3.2. Loneliness as Missing Out

#### 3.2.1. A Lack of Desired Relationships

Participants described how individuals experiencing loneliness often lacked a close group of friends at school, and saw themselves as having fewer friends than their peers.

“for example, if you are in class and everyone has a few groups of girlfriends and friends and you don’t belong to any group [hmhm] […]” (Belgian, female, 13 years).

Participants also talked about how someone experiencing loneliness might have a group of friends, but not feel like they fit in that group:

“being alone when maybe you don’t have any friends. Feeling lonely when maybe you have friends but it’s not that…that is you don’t feel included in the group” (Italian, female, 12 years).

“when you have no friends or when your friends play with each other and don’t invite you, you feel lonely” (Italian, male, 12 years).

Participants reported that someone might feel lonely when they are actively excluded by their peers:

“[they feel lonely when] others exclude them, let’s say from the group, from the games…like if I talk to you, I exclude another girl, she feels lonely” (Italian, female, 9 years).

“if your friend was maybe playing with another friend on the playground and you weren’t allowed to play” (Belgian, female, 8 years).

“erm, if he sends, for example, to a friend ‘can we meet up’ but they constantly ignore them […]” (Belgian, male 13 years).

#### 3.2.2. Difficulty Connecting with Others

Participants talked about how those who experience loneliness might have trouble developing relationships with others due to trouble communicating, particularly with peers.

“I think they see people who they like, but are afraid to talk to them. because they are there, because they fear they will not like that person or that they will have a negative influence on you” (Belgian, female, 13 years).

They reported that someone experiencing loneliness might not feel comfortable socialising with others:

“[…] not daring to say much in a group project because you don’t feel comfortable” (Belgian, female, 14 years).

Participants reported that someone experiencing loneliness might spend time away from others. That may prevent them from building connections.

“if there are a lot of kids playing and you sit there alone. Someone who is lonely would just sit there. […]” (Belgian, male, 8 years).

Italian participants described how someone experiencing loneliness might withdraw in order to protect themselves from the negative emotions that they associated with attempting to connect with others:

“they stay aside because maybe they don’t want to be heard in the group…and they think that nobody wants them” (Italian, female, 12 years).

“when they feel lonely, they feel sad so, they try even more to be alone to avoid being even sadder. They isolate themselves” (Italian, male, 12 years).

A lack of support. Participants talked about how someone experiencing loneliness might not feel like they are supported by those around them, meaning that they had to deal with the difficulties they faced on their own.

“It’s not having anyone, not having support, not having a person to rely on” (Italian, female, 12 years).

That lack of support could be reflected by conflict in relationships where that support would come from, such as with peers and parents.

“Maybe when their parents yell at them because they did something they shouldn’t have done, and they think they are alone. When their parents actually love them because they are their children” (Italian, male, 12 years).

### 3.3. Loneliness and Thinking Negatively

#### 3.3.1. Thinking Negatively about One’s Self

Participants reported that loneliness would often involve thinking negatively about one’s self. Someone experiencing loneliness may consider how the way they act might be causing difficulties in developing strong social connections.

“they think, because they are alone, if they are about to do something wrong or if they have done something wrong […] (Italian, male, 9 years).

Participants also reported that they might think they are being discriminated against by other people due to being different.

“maybe they think, also because of their character or physical appearance […] other people think they are different and then they tend to marginalise them and then they may feel bad” (Italian, male, 12 years).

In that sense, participants thought someone might blame themselves for their feelings of loneliness:

“that nobody likes him, and that it’s all his fault”(Belgian, male, 14 years).

#### 3.3.2. Thinking Negatively about Others

Alternatively, participants thought those experiencing loneliness might blame others around them for their experiences of loneliness. That involved feeling angry and jealous towards their peers and making comparisons between their lack of friends with others’ seeming successful friendships.

“Erm, for example being jealous that they are popular and have lots of friends [as opposed to you]” (Belgian, Male, 13 years).

“Angry at others. Why don’t they want to play with me now? Why can’t I play? Why can’t I make friends? Is it because of me or is it them?” (Belgian, male, 9–10 years).

### 3.4. Loneliness as a Transient Experience

#### 3.4.1. The Influence of Situational Changes

Participants primarily considered loneliness to be a temporary state that could change according to the situation a person was in. Participants thought that feelings of loneliness might vary across time and place. They thought there might be certain life events where loneliness might occur, such as moving to a new place or experiencing a bereavement:

“maybe you can still have friends, but still cannot say anything to them or often cannot do anything with them [hmhm] for example, those who are in another country or something, have moved” (Belgian, male, 13 years).

“for example, that he lives with someone else in the family because his parents have been killed in an accident or something, and that things go worse at school as a result” (Belgian, male, 14 years).

Participants noted that experiences of loneliness might occur when in conflict with those around you:

“with me, when I’m lonely, in that one big fight, I usually go to the toilet cubicle. When I feel lonely it is usually because everyone is against me even if there are some on my side” (Belgian, male, 9–10 years).

Perhaps once that conflict was resolved, loneliness would dissipate:

“[…] sometimes when I have a fight we don’t play for a while. When the fight is over, you don’t feel lonely anymore” (Belgian, female, 9–10 years).

Loneliness takes place at school. Loneliness on a day-to-day basis was seen to primarily take place at school, where young people navigated relationships with their peers:

“usually, if I am sad then yes. That is not at home, but at school, I always go to the toilet in the cubicle and then I sit there alone. That is really only when it is bad” (Belgian, male, 9–10 years).

It was thought that someone might feel better at home where they had their parents, they were comfortable around:

“maybe they are more lonely at school because they have no friends, but not at home because they get along well with their mum and dad” (Belgian, female, 8 years).

However, conflict with those at home was also mentioned as a trigger for loneliness, particularly for younger participants:

“If you are excluded or if you are not allowed to participate somewhere. Or if my sister, mom and dad are angry with me. Then I start to feel lonely.” (Belgian, male, 8 years).

#### 3.4.2. Loneliness According to Gender

Participants generally thought loneliness would be felt in the same way whether male or female:

“because we are all human beings anyway and I don’t think there’s much difference” (Italian, female, 12 years).

However, some participants explained that the way someone dealt with loneliness might differ according to gender.

“If the boys are bullied, they sometimes get angry and ask for a few boys. If they are angry with each other then they ask those boys to attack them; and girls, if they are excluded then go alone or play with someone else” (Belgian, female, 9–10 years).

“[…] boys don’t show their feelings as much, much less than girls, so maybe it is more noticeable with girls than with boys” (Belgian, female, 14 years).

#### 3.4.3. Loneliness Changes as You Age

Italian participants talked about how loneliness may become more prevalent as someone gets older due to changes in the way we experience our emotions and interpret the world:

“maybe when we grow up, we feel new emotions” (Italian, female, 12 years).

“children have another way of feeling things, a child’s heart is much more fantastic than an adults, because it develops, becomes smarter and you forget the things you like” (Italian, male, 9 years).

When presented with different scenarios in which someone might feel lonely, some participants reacted differently according to age. Younger participants tended to put more emphasis on the importance of having friends to play with, and not wanting to share their secrets with others:

“because you have no one here to play with. I think that’s worse than telling my secrets [not being able to tell secrets].” (Belgian, male, 8 years).

“because it is not quite the same. Because telling secrets is harder. Those are things that you should really not tell. When playing it is a child who just wants to have some fun and then that is something else” (Belgian, male, 10–11 years).

Participants who were older saw more value in being able to share your secrets with others than having people to play with:

“I personally think not being able to share your secrets with anyone [is worse]. Because you sometimes need that, otherwise everything will be bottled up like that. [hmhm, yes] into one sphere.” (Belgian, male, 13 years).

“If those, like that, are really really deep secrets that you really can’t talk to anyone except that one person you miss, [hmhm] I think you can feel more unhappy, because then I guess you can still do nice things with people, but you don’t tell them everything” (Belgian, female, 13 years).

However, it is important to note that participants did not talk about loneliness in those ways until prompted, and that some participants did not consider that there would be any differences between the two types of loneliness experience.

### 3.5. Coping Strategies That Aim to Alleviate the Negative Emotional Impact of Loneliness

#### 3.5.1. Positive Alone Time

Participants suggested strategies that someone experiencing loneliness could engage in to ameliorate their feelings of loneliness. Participants suggested someone could engage in activities that did not require the involvement of other people in order to be enjoyable. They made suggestions such as going for a walk, cycling, playing a computer game, creating artwork, reading, spending time on your mobile phone, or watching TV.

“they might like to go buy themselves some games and play with them, so they don’t have to think about it” (Italian, male, 12 years).

#### 3.5.2. Expressing Thoughts for Themselves

For young people who might not have someone to talk to, participants suggested they might find comfort in writing down their thoughts. That was suggested primarily as a way in which they could express themselves without the need of a confidant:

“For example, write in a book, write down thoughts like that, or draw or go cycling or walking [yes] to be able to do something”.

They considered that writing in a diary might be a safer way to express yourself rather than revealing secrets to your peers:

“He has to write his secret in his diary. Then he is not concerned anymore. And then the problem has actually already been solved. He should not tell other children because some people cannot trust you if you do not yet have one. He’s not allowed to say that. Or if I’m alone, say it out loud to get it out” (Belgian, female, 9–10 years).

They also suggested this could act as a starting point for opening up to someone, if they were unsure of how to express how they were feeling in words:

“gosh for example, making a drawing or something in which you express your feelings and then show it to that adult” (Belgian, male, 13 years).

#### 3.5.3. Parents as a Source of Comfort

Participants suggested that parents could be a source of support for someone experiencing loneliness. They reported that parents could act as a confidant, particularly if the person had no peers to talk to about their secrets or did not want their peers to know about their secrets:

“if he really doesn’t have anyone to talk to, he might as well talk to his parents, who would definitely listen to him” Italian, male, 12 years).

They talked about how parents could offer advice and encouragement, but also how parents could often just be a source of comfort by being close by or providing physical affection:

“I think, especially as a parent of a child [hmhm] and when you know that your child feels lonely, you really pay attention to that he or she doesn’t feel lonely at home. By giving them a lot of attention, or by doing fun things with them. And also have a chat, talk with them” (Belgian, female, 14 years).

### 3.6. Coping Strategies That Aid Social Reconnection

#### 3.6.1. Changing the Way You Act

Participants suggested ways that someone might overcome loneliness that involved reconnecting with those around them. Participants recognised how an individual’s willingness to make effort with others might be important for determining if they were successful in doing so. They suggested simply asking to join in with a group,

“try to make friends, talk to them, invite them, take initiative” (Belgian, male, 14 years).

“make more friends and always make sure you invite someone over to do something, and if they can’t, ask someone else so that if you really want to do something you can still do” (Belgian, female, 14 years).

Also, by trying to be more open with peers:

“try to always be yourself […] try to open up a little bit more, but not too much because if not afterwards maybe you can seem too exuberant or too impulsive and […] not too much or too little, that’s the middle ground” (Italian, female, 12 years).

“maybe if they have a class to go and talk to someone, maybe even in group work expose themselves and not stand there in silence and look at the void” (Italian, female, 12 years).

However, they recognised that there might be some challenges in making new friends,

“Not easy to make new friends because you are lonely. Then you may not know how to address someone” (Belgian, female, 8 years)

“No [not easy] because you were just thinking ‘that won’t work anymore’. Then I just won’t ask that anymore [to make friends]” (Belgian, male, 8 years).

#### 3.6.2. Peer Efforts toward Inclusion

Participants also suggested ways that those around someone experiencing loneliness might help to make them overcome loneliness. They suggested that peers approaching them, as opposed to relying on the person experiencing loneliness to make the effort, might be beneficial:

“go there to the lonely person and…invite them to come with me […] I would play with them, take away the sadness they have!” (Italian, male, 12 years).

“yes, go and talk to them, and say that that person can join you and sit with you and stuff” (Belgian, female, 13 years).

They suggested peers might organise an activity for a group to do together including the person experiencing loneliness:

“um for example at work, for example having a coffee together during the break, or just cycling along in the morning or something” (Belgian, male, 13 years).

#### 3.6.3. Teachers Encouragement of a Cohesive School Environment

Participants suggested teachers could take on the role of encouraging socialising in school such as implementing more tasks that involve group work:

“yes, I think if the person in the classroom sees that someone is alone or something, that they will put that person with other people, such as working more often in groups so that that person can also make friends” (Belgian, female, 14 years).

Teachers can also act to solve problems by talking to an individual about how they feel, and encouraging peers to include them,

“there we had to take off our shoe and put it with our group of friends. And then the teacher said, ‘are we going to try a little bit to play with Marie’” (Belgian, male, 9–10 years).

“tell the teacher that she feels lonely and no one wants to play with her. Then the teacher can do something about it, talk to she feels a bit more secure” (Belgian, female, 10–11 years).

### 3.7. Secondary Analysis to Explore Gender Differences in Coping Strategies

Initial TFA identified that participants had mixed ideas about whether gender influenced experiences of loneliness; generally, it was agreed that the way loneliness felt would be the same irrespective of gender, but that how it was dealt with may differ. It may be assumed that participants suggestions on how to cope with loneliness may differ according to whether they are a male or female. To further investigate, a deductive content analysis was conducted to compare the differences between the most frequently suggested coping mechanisms according to gender. Male and female participants made suggestions for certain coping strategies in regard to the lonely person and the context at similar rates. The most common suggestions involved someone feeling lonely talking to family and making new friends. In terms of suggestions for those around young people, the most common was for peers to include them. Participants differed slightly by gender in suggestions for peers to listen/talk to someone feeling lonely, with male participants (50%) more likely than female participants (16.67%) to make that suggestion. That is contrary to participants’ discussions of gender in relation to loneliness experiences, in which some suggested boys may be less willing than girls to talk about how they were feeling, and that it was easier for girls to do so. Perhaps, boys find it difficult to instigate conversations about their feelings, but would welcome that conversation if instigated by peers instead. Female participants (58.33%) were more likely than male participants (33.33%) to suggest that someone feeling lonely to engage in coping strategies that involved being more sociable. That fits with girls’ ability to talk to others about their feelings more freely than boys: female participants assume that making more of an effort to be sociable would be received positively from peers.

### 3.8. Secondary Analysis to Explore Age Differences in How Experiences of Loneliness Are Conceptualised

Previous research theorises that, because friendships take on different roles across the lifespan, loneliness will be experienced due to different social contexts at different ages [28]. Initial analysis in the current study identified potential differences between ages in regard to how loneliness was conceptualised. To further investigate, a deductive content analysis was conducted to examine age differences in the social contexts for loneliness (having no one to play with or having no one to share secrets with). In the interviews, participants were told about loneliness as a result of not having anyone to play with, and loneliness as a result of not having anyone to talk to about their secrets, and asked which, if any, they considered would be worse to experience. This part of the secondary analysis utilised only the data from Belgian participants, because Italian participants had a smaller range of ages to compare (up to 12 years).

Results show that 8- to 11-year-olds (66.67%) were more likely than 12- to 14-year-olds (33.33%) to report that not having people to play with would be a worse reason for loneliness than not having someone to talk to about secrets. In congruence, 12- to 14-year-olds (66.67%) were more likely to report that it would be worse to feel lonely due to not having someone to talk to about your secrets than 8- to 11-year-olds (16.67%). In terms of reasons for their choice, 8- to 11-year-olds were more likely to report that they prefer having someone to play with over sharing secrets (66.67%; 16.67%), whereas 12- to 14-year-olds were more likely to report that it was important to have someone to talk to (66.67%; 0%). The results support the idea that the antecedents of loneliness may change as you age, depending on the roles of friendships, with friendships taking on the role of playmates in childhood, and transitioning to include more emotional support in adolescence.

## 4. Discussion

The current study explored how young people conceptualised loneliness, whether they considered there to be differences in loneliness according to gender, and the strategies that they considered would be useful to help someone overcome loneliness. There is currently little qualitative research that investigates the meaning that adolescents give to loneliness; the current study adds to the knowledge base by utilising a systematic in-depth qualitative analytical method, TFA, that ensures consideration of the dataset as a whole to develop themes that provide a narrative about the participants’ perspectives. Participants described loneliness as a negative, but transient experience that primarily took place at school, and was caused by feeling excluded. Participants suggested coping strategies that focused on ensuring inclusivity at school might help to alleviate loneliness.

### 4.1. What Young People Thought Constituted Loneliness and What Did Not

Participants identified that loneliness was a negative emotional experience that occurred when a person felt like they were disconnected from others; it was often described as involving sadness, reflecting the findings of previous qualitative work with adolescents [23,29]. It was seen as different to being physically alone because someone could be physically alone, but still have the knowledge that they had people in their lives who supported them; someone experiencing loneliness would experience negative cognitions about their relationships with others, and feel unsupported. Previous work identified young people’s conceptualisations of loneliness to be multi-faceted, including (a) the existence, or lack of meaningful friendships, and (b) their attitudes towards being alone [23]. In relation to the current study, friendships with peers were purported to be central to loneliness experiences, but young people focused on the experience of being excluded as a reason for loneliness, as opposed to the meaning that was given to those friendships. That may reflect how for those younger participants, childhood friendships were focused on proximity and companionship, as opposed to the intimacy which characterises friendships in older adolescence. The findings also suggest that the presence of negative friendships, i.e., those where the individual feels excluded, not just the absence of positive friendships, may be an important facet of adolescent experiences of loneliness. In terms of being alone, participants thought that attitudes towards being alone may be more negative for those experiencing loneliness than those who are not. That was because, for those who were experiencing loneliness, they would feel aloneness physically, but also feel mentally disconnected from others; whereas those not feeling lonely would be aware that any negativity felt whilst alone would dissipate once in the company of others. Those findings can help to explain why physically being alone is often associated with loneliness.

### 4.2. Gender Differences in Loneliness

We found that participants regarded feelings of loneliness to be the same regardless of gender: boys and girls would feel equally negative emotions. However, participants reported that they believed the way someone reacted to loneliness would differ according to gender. Some participants believed that boys would be more likely to hide feelings of loneliness than girls. They believed that for boys, friendships focused on participating in physical activities together, whereas for girls they would spend more time talking. In that sense, they thought it would be easier for girls because they would be more likely to talk about how they were feeling. That is reflected in research on gender differences in friendships that show girls’ friendships have higher levels of peer support, and boys’ friendships have higher levels of conflict [30].

Previous qualitative research has not focused on whether young people conceptualise loneliness differently according to gender, but a recent meta-synthesis of qualitative research on depression and loneliness in young people aged 11 to 30 years did not identify any thematic patterns according to gender [9]. A recent meta-analysis of gender difference in loneliness across the lifespan found a small but significant effect, with males experiencing loneliness more prevalently than females in childhood and adolescence. The meta-analysis found no differences in gender according to whether loneliness was due to desiring more intimacy, or more relationships which reflects current participants’ beliefs that loneliness is experienced in the same way irrespective of gender. Those findings suggest that in terms of how loneliness is conceptualised, gender has little impact. However, when thinking of ways to address loneliness in young people, considering how gender differences influence the dynamics of youth friendships might help in developing strategies that can be implemented irrespective of friendship dynamics. For example, creating support groups made up of classmates from different friendship groups could help those feeling lonely to receive support without having to ask their friends, and provide the opportunity to develop new connections.

### 4.3. Types of Loneliness

Weiss’ typology of loneliness posits that loneliness can be broken down into: (1) social loneliness, in which a person desires a higher quantity of social connections, and (2) emotional loneliness, in which a person desires more intimate connections [10]. In early adolescence (aged 12 years), loneliness has been found to negatively relate to number of friends, and for friendships they do have, those experiencing loneliness rate those friendships less positively than their friends [31]. We would expect then that participants in the current study would relate more to social loneliness, than emotional. The two loneliness types were described through the use of short vignettes in which a young person (a) desires more people to have fun with, or (b) a young person desires someone to share their secrets with and illustrated with picture cards depicting those scenarios. It was clear that participants did not think about loneliness in two distinct types: they tended to relate more to either description depending on their age. Younger participants (below 12 years) valued having friends to play with, and generally felt uncomfortable about the notion of telling someone their secrets; on the whole, older participants related more to the emotional impact of not having someone to share secrets with. For younger adolescents, friendships may be seen as serving the function of companionship, and parents serve the role of emotional support meaning that emotional intimacy is not a prerequisite for friendship [32]. Older adolescents recognised the emotional difficulty of not having someone to share your secrets with rather than to have fun with, although they did also recognise that would be a difficult experience too, albeit less so. During the transition into older adolescence, youth seek autonomy from parents, begin to build their self-identity, and rely more on peers for emotional support: self-disclosures emerge as a feature of friendships [28,33]. The current findings suggest that the relationship between loneliness and friendship during adolescence may change as youth move through developmental stages. Indeed, some participants stated that they believed loneliness would become more prevalent with age, because emotions and ways of relating to others become more complex.

However, labelling loneliness according to a certain type depending on the age an individual appears too simplistic; there were instances where younger participants valued having someone to share secrets with, and older participants valued having people to have fun with. That may be attributed to the different rate at which individuals move through developmental stages [34], but it could also be that for adolescents, the two distinct types of loneliness do not fit with the dynamics of their relationships. For instance, for younger adolescents intimate connections are not defined by disclosures and emotional support, but instead by companionship. Therefore, emotional loneliness is not absent, but instead exists in a different form than for older adolescents. For example, it might look like having someone to participate in the games you like to play, rather than having someone who shares the same values and beliefs as you.

### 4.4. Loneliness and Difficulty Connecting with Others

Participants thought that those experiencing loneliness would have trouble establishing fulfilling relationships with peers; that they would feel as if they did not fit in friendship groups that existed for their peers. Participants thought that difficulties might exist due to individuals being afraid to talk to their peers, but also that difficulties might occur due to isolating themselves to manage negative emotions associated with socialising. In some cases, participants reported that thoughts about being disconnected might occur even when an individual had people who could be there to support them. That reflects Cacioppo and Hawkley’s model of loneliness by which a lonely person distances themselves from potential relationships due to their negative social cognitions, paired with experiences of negative behaviours from others that match their negative expectations [35]. Indeed, some participants indicated that those experiencing loneliness would blame others, citing anger and jealousy as the emotions they would feel towards others that would again reiterate Cacioppo and Hawlkey’s model [35]. Some participants reported that those experiencing loneliness would harbor feelings of self-blame, and focus on their characteristics as reasons why others did not like them. Contrary to previous work that has focused on deficits in social skills as a reason for loneliness, participants in the current study mentioned ‘unattractive’ physical appearance, and a sense of feeling disliked due to being different. Our findings combined with those of Jenkins et al. who argued that feeling excluded from others may be related to a variety of external factors, such as belonging to a marginalised group, that are beyond individual control, argue that the current focus on social skills as a reason for disconnection may be misplaced [29].

### 4.5. Situations in Which Loneliness Occurs

A novel finding from the current study was that participants considered loneliness to be a transient experience that could be dependent upon certain circumstances and situations. Conflict with those close to you was considered to be a trigger for loneliness; accordingly resolving conflict could lead to loneliness dissipating. On a day-to-day basis participants situated loneliness as taking place at school, they suggested it would be alleviated once the individual was home and could seek comfort from their parents. That reiterates how it is important to acknowledge the contexts in which loneliness occurs, being in a supportive environment can help to alleviate feelings of loneliness and equally, a hostile environment can give rise to loneliness. In congruence with longitudinal quantitative research with adults, certain life events such as moving to a new place, or experiencing family changes were considered to be triggers for loneliness [36]. Adapting to those new circumstances meant that a young person could overcome loneliness. That differs from previous qualitative research in which young people reported themselves to feel stuck in their experiences of loneliness [23]. Knowledge that loneliness is temporary may, therefore, be beneficial in overcoming it; indeed, qualitative research with adults found that knowledge that loneliness is temporary was given as a reason by those who considered it to be positive [37].

The situations in which loneliness was considered to occur reflect times in which young people felt disconnected from others in their social environment, such as when moving schools or experiencing mistreatment by peers. Research identified in the grey literature reiterates those situations, but identifies additional situations that young people consider could trigger loneliness; they include loss of significant relationships, experiencing mental health challenges, living with disability, and practical barriers to socialising with peers, e.g., access to transport [38]. However, that research includes participants from a wider age range, 10–24 years, and the methodological process is unclear. It is possible that these additional situations were reported by the older adolescent participants, and may not be applicable to younger adolescents such as those in the current study.

### 4.6. Overcoming Loneliness

Participants suggested ways that loneliness could be overcome by young people. They thought strategies that did not require other people, such as writing in a diary or engaging in an enjoyable activity could help those experiencing loneliness to express or distract themselves. Previous research with youth experiencing loneliness identified distracting activities as their main source of coping, but such strategies were only able to provide short-term relief [23]. They may be useful when combined with more long-term coping strategies such as those that aid social reconnection. In the current study, participants thought someone experiencing loneliness might benefit from making a greater effort to be open to socialising with peers; they described those who experience loneliness as spending time away from others, suggesting that participants considered loneliness to be partly due to the individual’s personality, or behaviour. That reflects the previously identified general stigma towards those experiencing loneliness, particularly when they are also reclusive [39,40]. Holding stigmatising views about loneliness can be detrimental for young people, because they are likely to internalise that stigma which impacts negatively upon self-esteem [41]. Interventions that reiterate how loneliness is a normative experience for adolescents, and provide an understanding about why loneliness happens might help adolescents who are attributing their experience of loneliness to their personal characteristics to understand that it is not an issue specific to only a certain ‘type’ of personality, i.e., introverted.

Participants made suggestions for how those around an individual experiencing loneliness might help. Suggestions made for both peers and adults centred around creating opportunities for those experiencing loneliness to develop social connections. Peers could help by approaching the individual to spend time with them, or ask them to join in with play; in doing so the pressure to be included is taken off the individual experiencing loneliness’ shoulders. Teachers can help to facilitate those interactions by creating opportunities for inclusion in the classroom, such as through group work. In consideration of the current findings, school-based interventions are recommended for youth loneliness. Interventions that are whole-school based, and universal could help to alleviate the stigma of loneliness by ensuring individuals are not singled out as lonely. A school ethos that focuses on inclusionary behaviour, with opportunities to enact those behaviours, could help develop an inclusive environment, creating a community of individuals that trust and care about each other. Participants suggested strategies such as group work could be promoted within schools to encourage socialisation between peers; such strategies have the advantage of already being familiar to teachers, and do not require changes to the existing curriculum in order to be implemented.

### 4.7. Measures of Loneliness

Current measurements for adolescent loneliness fail to consider how the conceptualisations of loneliness may differ from that of adults. The majority of measures for adolescent loneliness are based upon measures of adult loneliness, as opposed to adolescent conceptualisations of loneliness, and we need to address that mistake. That is important because the contexts of adolescence differ from those of older adults; older adults are likely to experience social isolation due to bereavements, mobility issues, unemployment, whereas adolescents are surrounded by people at school or in work. In the current study, adolescent loneliness was situated within school. Links between loneliness and school connectedness have been identified in previous quantitative research [42]. That suggests measures for adolescent loneliness should make contextual specifications. Items could be included such as “I have friends (a) at school, (b) outside of school, (c) online” to ensure that young people consider different contexts when they complete measures. The UCLA [43] is one of the most widely used measures for loneliness, often being used in research with adolescents, and includes items recommended by the ONS for use with children [2]. Currently, the UCLA [43] includes items about social relationships more broadly, “people are around me but not with me”, “I have nobody to talk to”, “no one really knows me well”. For a young person, the reaction to those sentiments may differ contextually: they may feel supported at home whilst unsupported at school. The lack of specificity in adolescent loneliness measurements mean such nuances are overlooked.

The Peer Network and Dyadic Loneliness Scale (PNDLS) [44] is a commonly used measure for adolescent loneliness; its development was based, in part, on Weiss’ (1972) [10] typology of loneliness. In the current study, participants did not recognise loneliness as relating to social or emotional typologies, instead both were intertwined. To measure peer dyadic loneliness, the PNDLS includes items such as: “some kids don’t have anyone special their age to share things with”, “some kids don’t have a friend that they can talk to about important things”. Based on the current findings, such items may wrongly identify younger adolescents who prefer not to share secrets, as lonely.

The Perth-A Loneliness scale (PALS) [45] is a lesser used measurement of adolescent loneliness, but one that has included the voices of adolescents during development. The scale has primarily been used in research with Australian adolescents, but items align well with the findings of the current study. The items cover attitudes towards friendship (“my friends will stand by me in almost any difficulty”, attitudes towards solitude (“I have discovered the benefits of being alone”) and experiences of isolation (“I have nobody to talk to”), which are all reflected in the current study. However, the scale misses items to account for the presence of negative friendships that were identified to impact experiences of loneliness in the current study, and in other qualitative work [46]. Additionally, the scale could benefit from including items to identify negative cognitions; participants often reported that someone experiencing loneliness would think negatively about themselves particularly in regard to their social capabilities, e.g., “I am not good at making friends”, “I am not sure that my classmates like spending time with me”, “I do not think that people find me fun to be around”. Nevertheless, the scale is a promising measure for adolescent loneliness, and the current study suggests it may be suitable to use with adolescents outside of Australia.

### 4.8. Strengths and Limitations

The current study was able to capture the voices of adolescents who were likely to have had a range of different experiences due to differences in age, gender and country of origin. The study recruited participants from Italy, and Belgium two places with cultural differences: Italy is considered to have a collectivist culture, whilst Belgium is considered to have an individualistic culture. Through the use of TFA, the study was able to identify both similarities and differences in the ways adolescents conceptualised loneliness. That led to overarching themes that relayed a nuanced narrative about adolescent loneliness, for example, Italian participants were more likely to describe loneliness as something that comes from within, whilst Belgian participants were more likely to describe loneliness as a result of external factors such as being excluded. Recognising those nuances can help us to develop interventions that address the whole experience of loneliness, as opposed to its components. Whilst those findings indicate differences in conceptualisation according to culture, the timing of data collections differed between the two countries meaning that the Belgian interviews were conducted prior to COVID-19 social restrictions, and Italian interviews were conducted during. Therefore, it cannot be established that such differences are not due to the impact of the pandemic, with those interviewed during the pandemic more likely to have experienced social isolation, and disruptions to relationships. The difference in data collection timings meant that the study benefited from including participants who had a broader range of experiences that may have impacted their understanding of loneliness, than those who have not experienced social restrictions. However, further research is needed to investigate differences in conceptualisations between cultures.

The study is potentially limited by not identifying whether or not the participants had, or were, experiencing loneliness. Instead, the interviewees were asked to talk about loneliness from a third person perspective. That way, participants could share their thoughts about loneliness without having to share personal experiences which overcome potential distress and reluctance to talk that can occur when asked to talk about experiences of loneliness [47]. In addition, it is likely that coping strategies suggested by young people are the same that they would use if they were to experience loneliness, and so we were still able to identify strategies adolescents consider useful for coping with loneliness

## 5. Conclusions

The current study investigated adolescents’ conceptualisations of loneliness, including how to cope with it. TFA was used to develop six themes that describe the salient issues relayed by participants. The findings identified that for adolescents, loneliness was considered to take place within school, and centred around relationships with peers. Such contexts should be taken into consideration by those measuring adolescent loneliness because adolescents may differ in whether they consider themselves to be lonely depending on the context they are asked about, i.e., home versus school. There also appeared to be developmental differences in loneliness conceptualisations: younger adolescents identified more with the difficulties regarding lacking friends to have fun with, whereas older adolescents identified more with the difficulties of lacking someone to share their secrets with. Findings indicated that loneliness could not be attributed to one cause, but instead could involve negative cognition about social relationships; experiences of conflict and being excluded; changes in circumstance such as moving to a new place; and social withdrawal. Measures of adolescent loneliness should ensure that peer relationships are addressed clearly by items, and pay attention to how desired qualities for friendships may differ according to age with younger adolescents focusing on companionship, and older adolescents focusing on emotional support. The Australian measure for adolescent loneliness, “PALs”, includes items that reflect adolescent conceptualisations better than more commonly used measures, like the UCLA. The current findings provide some support for its use with adolescents outside Australia. Suggestions on how to overcome adolescent loneliness included increasing opportunities to become included by peers. Interventions for adolescent loneliness should utilise strategies that promote inclusive school environments.

## Figures and Tables

**Figure A1:**
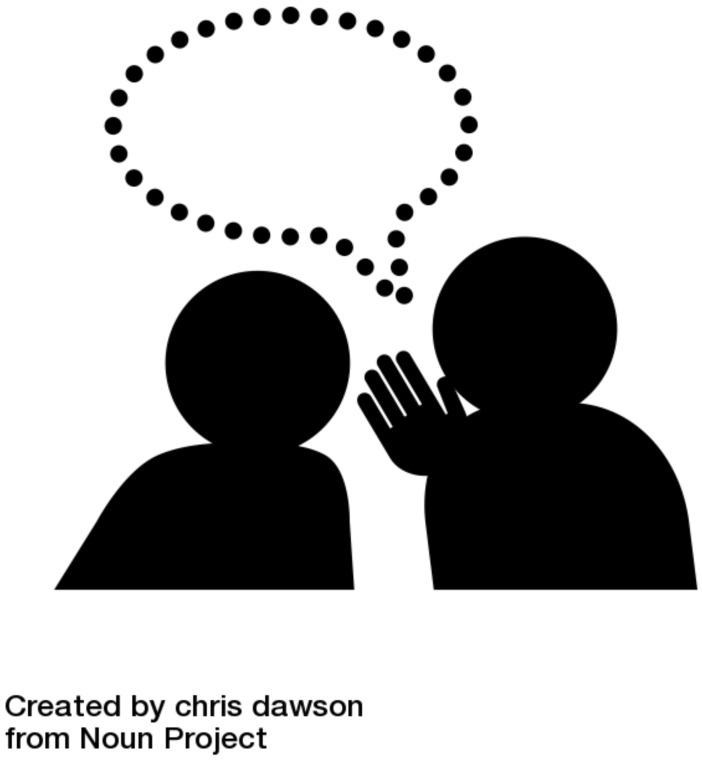
Emotional loneliness.

**Figure A2:**
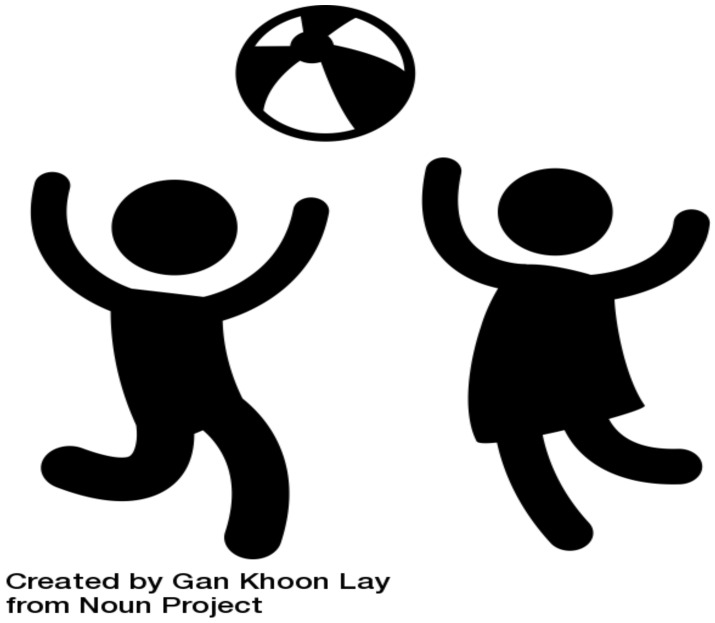
Social loneliness.

## Data Availability

The data presented in this study are available on request from the corresponding author. The data are not publicly available due to confidentiality.

## Appendix A

Interview Protocol

Interview Protocol for Children 8–11 years

Intro:

- Putting the participant at ease (familiarise the participant with the interview setting):
  ○ “What class did you just have?”
  ○ “Did you like the class/lesson?”
  ○ “What subject are they teaching here?”
  ○ “I can see you looking around, do you know this room?”
  ○ “Do you know why I am here?”
  ○ “Thank you for taking the time to think about the questions I am about to ask you. Your parents have agreed to allow you to talk with me.
- Go over the participant information sheet with the child and give them the opportunity to ask questions. When the child consents and wants to participate in the study, ask them to sign the consent form.

− General information about confidentiality:
  ○ “Before we start, I want to tell you some important things. I am going to ask you some questions, which you may answer. You may recognise that this is called an interview. Like the people on television that ask questions. You can stop with the interview whenever you want, and you don’t need to tell me why you want to stop. It’s no problem if you want to stop or take a break. Also, only me and the other researchers of this study will get to know your answers. We will not tell your answers to anyone else, unless we think you might be in danger.”
  ○ “To keep your answers private, we should choose a pretend name to call you, shall we choose that together now? Or I can choose one for you…”

• Audio recorder:
  ○ Putting the participant at ease: Perhaps try out the recorder, record something and listen back to it.
  ○ “When I use the audio recorder, I can look at and listen to you much better. After our conversation, I can re-listen to everything very carefully.”
  ○ “I am learning myself. It is possible that I ask some weird questions. You can tell me if you find the suggestions a bit strange.”
  ○ “When I re-listen to our conversation, I can learn about what you told me and get to know more about loneliness and write up the conversation. The conversation gets deleted 4 months after it has been written up.”

− Start:
  ○ “Are you a bit nervous?”
    ■ Yes: “That is no problem and very normal. A little bit of nerves can help us to think better. I think it’s good to hear your ideas, opinions and experiences so there are no right or wrong answers, all answers are good ones. Taking part in the interview may help other boys and girls your age.”
    ■ Is there anything else you’d like to know before we start?”

Main questions:

− The focus of our interview today is going to be “loneliness”. First let’s start by imagining you meet a group of aliens who have just landed on Earth. These aliens don’t know very much about life on Earth. They don’t know how and what a person feels and thinks. Can you explain to them what loneliness is?
  ○ “Thanks for telling this so comprehensive!”
  ○ “You have given me plenty ideas about the words we can use to explain loneliness.”
  ○ “Thanks, those are a lot of new words, ideas and examples.”
  ○ “Now I can explain loneliness much better to the aliens. Thank you!”
  ○ “Would you tell the aliens if there is a difference between boys and girls who feel lonely?”
− How do you think someone, who is lonely, feels?
− How do you think someone, who is lonely, thinks?
− How do you think someone, who is lonely acts?
  ○ “Why do you think he/she…”

• When do kids feel lonely?
  ○ “What can happen when kids feel lonely?”
    ■ (to see if they recognise the different types of loneliness and ask further questions)

− Researchers would tell the aliens that there are two types of loneliness. Some children feel lonely because they don’t have anyone to tell everything to or share secrets with. Other children feel lonely because they don’t have a lot or any friends to play with.
  ○ “Imagine, a child feels lonely because he/she doesn’t have someone they can tell everything to or share secrets with. How would he/she feel?
  ○ “Imagine, a child feels lonely because he/she misses other children to play or have fun with. How would he/she feel?
  ○ Depending on the answer:
    1. “Okay, so you think both children feel the same. I hear both, sometimes they feel the same sometimes they feel different.”
    2. “Okay, so you think both children feel different. I hear both, sometimes they feel the same, sometimes they feel different.”
    3. Asking further: examples, answers…

• If someone in your class feels lonely:
  ○ “What kind of advices/tips would you give him/her?”
  ○ “How would you help him/her?”
  ○ “Think about the child who feels lonely because he/she had no one to tell everything to and to share all their secrets with. What could he/she do to feel less lonely?”
  ○ “Think about the child who feels lonely because he/she doesn’t have/know enough friends to play with. What could he/she do to feel less lonely?”
  ○ “You gave them both the same/different tips? Would you like to explain this a bit? What do you think is the biggest difference between them?”

Finishing the interview:

− “What did you think of the interview?”
− “Did you think it was difficult?”
− “What did, or didn’t you find fun to talk about?”
− “What did you find interesting?”
− “Do you have some tips for me to talk to the other children?”
− After care: follow-up letter
− Thanking the child:
  • “Thank you for participating/helping by doing this interview”
  • “Here is a small gift as a thank you”
− Guiding the child back to the class:
  • “What class do you have now?”
  • “What are your plans for this afternoon?”

Prompting questions to use throughout the interview:

− Prompting neutral:
  • “Can you tell me some more about this?”
  • “Can you give me an example of...?”
  • “Could you show me?”
  • Repeat parts of their sentences for repeat some words very calmly while looking at them interrogatory.
− Prompting to convince them:
  • “You are a very important source of information. You can help other children by answering these questions.”
  • “It is very nice that you wanted to free some of your time so we can discover this together. This helps other children too.”
− Participant does not know the answer:
  • “That is fine, maybe something will later on…, you can always tell me then. I have another question for you.”
  • “There are no wrong answers”
− Ask further:
  • “I listened very carefully, and you said ‘…’, would you mind explaining this a bit further?”
  • “Can you explain this to me, a bit slower with more details? Like how you would describe a movie to me. You can draw it as a little comic, if you would want to/this can help you.”
  • “Did I get this right? Is this what you meant?”
  • ‘What’ and ‘How’ questions

Interview procedure adolescents (12–14 years):

− Ensuring that the adolescent feels at ease:
  • Where do you want us to sit?
  • Which class did you just have?
  • Do you know this (class-) room?
  • Do you know why I’m here?
− Go over the participant information sheet with the adolescent and give them the opportunity to ask questions. When the adolescent consents and wants to participate in the study, ask to sign the informed consent.
− Audio recorder: explain to the adolescent why recording the interview is useful and how it can help the interviewer later on.
  • When I use the audio recorder, I can really listen to you and to what you are saying.
  • It also helps me to hear what I say and do, and what I can do better. I’m still learning how to do this, so I might say silly things. If that happens, you can always tell me.
− Start:
  • Are you a bit nervous? (if yes; “That’s okay and very normal. It actually helps for thinking). Interviewer will stress that all answers will be good answers, and that he/she is interested in the ideas, opinions, and experiences of the child/adolescent.
  • Before we start, I want to tell you that I really need to dig deep into what you think and feel about my questions. I will be asking a lot of questions, even when you have already given me an answer. It’s important to me that you know that that is not because I didn’t like your previous answer. As I said before, all answers are good.
  • Your answers will not be shared with anyone, including your teacher, parents or family unless we think you might be in danger. To keep your answers private, we should choose a pretend name to call you, shall we choose that together now? Or I can choose one for you…
  • Is there anything you’d like to know before we start?

Main interview questions:

− Imagine someone were to ask you to write a dictionary. How would you describe loneliness?
  Would you say there is a difference for boys and girls?
− Make sure to acknowledge the difference between lonely and alone:
  • Make sure they understand this.
    ∞ Try to let them come up with an example of a situation where someone is alone but not lonely, and an example of a situation where someone is lonely but not alone.
    ∞ If they cannot do this, provide them with examples:
      • Someone who is gaming, could be gaming alone, but is not necessarily lonely.
      • Someone who is at a party is not alone, but could feel lonely, for example when they don’t really know anyone there or have no one to talk or have fun with.
  • You gave me many new ideas about what words we can use to describe loneliness.
  • Thank you; those are a lot of new words, ideas and examples.
− Someone who is lonely, how do you think he or she feels?
− Someone who is lonely, what do you think he or she thinks?
− Someone who is lonely, how do you think he or she acts?
  • How and what questions:
− When might someone feel lonely?
− Before, we talked about how you would describe loneliness in a dictionary. Some people would say there are two kinds of loneliness. Some people feel lonely because they don’t have someone who they can talk to about everything and with whom they can share their secrets. Other people feel lonely because they don’t have someone with whom they can do fun activities.
  • Say, someone feels lonely because they don’t have someone who they can talk to about everything and with whom they can share their secrets. How would they feel?
  • Say, someone feels lonely because they don’t have someone with whom they can do fun activities, like friends, neighbors or cousins. How would they feel?
  • Depending on the answer: You think these two people would feel the same? … that these people feel differently (ask further questions). … I understand that they sometimes feel the same and sometimes feel different,…
  • Try to get them to concretise their answers, let them give examples of possible reactions,…
− When someone in your class feels lonely,
  • What advice would you give them?
  • How could you help them?
  • Before we talked about how someone who felt lonely because they didn’t have anyone to say everything to and share his or her secrets might feel. What could they do to feel less lonely?
  • Remember we talked about how someone who felt lonely because they didn’t have anyone to do fun activities with might feel. What could they do to feel less lonely?

Finishing the interview:

- What did you learn or discover here today?
- What do you want to remember and what do you want to leave in this room?
- Do you have any tips for me? Can you tell me something I did well?
- Thank the adolescent:
  ∞ Thank you for helping by doing this interview
  ∞ As a thank you, here is a little present.
- Do you have any questions left? Is something bothering you?
- Reintegrate.
- What else are you going to do at school today?
- Which course do you have next? Do you like it?

Prompting questions to ask throughout the interview

− Prompting neutral:
  • Could you tell me more about that?
  • Could you give an example?
− Prompting persuasive:
  • You adolescents are an important source of information. You can help other children and adolescents with this.
  • It’s very nice that you took the time to help me and that we can go through this together. It will surely help other people.
− Give compliments through engaging communication:
  • Thank them, for specific behaviour, for a specific answer. Add that they contributed to your need, instead of rewarding them for “good behaviour”.
    ∞ Thank you, you explained that very clearly.
    ∞ I can tell you are really thinking about this, thank you.
    ∞ Thank you for telling me this much.
− The participant does not know the answer:
  • That’s no problem, maybe when you think about this later, you can still tell me. I have another question for you.
  • That’s no problem, maybe something will come to your mind in a bit.
− Asking supplementary questions:
  • I listened very well to what you told me, you said … can you explain that a little bit more? I want to make sure I really understand everything
    ∞ I listened very well to what you told me, but I don’t quite get it yet. Can you explain again for me, please?
  • Can you expand that for me, like you would describe a movie?
  • Do I understand this correctly? Is this what you mean?
  • What and how questions, to elaborate and to deepen, avoid why questions.
  • When asking supplementary questions about feelings, refer to them as someone else’s feelings, not their own.

Picture cards:

Picture cards were used to help illustrate the concepts of emotional, and social loneliness to participants during the interviews.

The interview protocol was devised by M.M and L.V. The main interview questions were asked in each interview, and the prompts were used where needed to help encourage the participant to expand on their answer, without leading them in any direction. The interview procedure was trialed and refined from previous versions into those described below. The original version included vignettes about a lonely boy (if the participant was male) or girl (if they were female) but the trials highlighted those scenarios to be confusing to the participant, and so they were simplified to “imagine a child/someone”. The interview protocol acknowledges important nuances regarding the concept of loneliness, i.e., the difference between loneliness and aloneness. Picture cards were used to help illustrate questions regarding types of loneliness.

## Appendix B

**Thematic Framework Analysis**

To analyse the data, TFA developed by Ritchie and Spencer [22], was utilised. TFA is comparable to thematic analysis in that it produces salient themes to reflect a narrative about the data, but includes additional stages that ensure robustness [48]. It is particularly useful when working in research teams because the systematic nature of the method ensures all team members can participate in each stage required in the analyses without losing track of the process. The inclusion of the coded data chart (i.e., the framework matrix) enables a reflexive approach to be taken in which theme development that involves continual reappraisal of coded data and ensures that all data, including deviant cases, are taken into consideration to avoid reiterating pre-existing biases about the research topic [44]. TFA includes seven stages, illustrated in Table A1, alongside how each step was realised in the current study.

**Table A1:** Using thematic framework analysis (TFA) to analyse the interview data from Belgium and Italy.

Stage	Description	Approach
1. Transcription	Transcribing the audio recordings into text files.	Interviews were conducted in the interviewees own language (Dutch or Italian) and then transcribed. Those transcriptions were translated into English in order for the UK team to be involved in analysis. Idiosyncracies in translations were checked and corrected by members of the research team who spoke both English and Dutch or Italian.
2. Familiarisation	A process of becoming familiar with the data set.	The UK team read and summarised all transcripts, including listing potential initial codes; the Belgian team did the same for the Belgian transcripts. Both teams came together to discuss that process and any initial patterns in the data.
3. Open coding	Developing an initial set of codes to be trialled and refined.	The UK team created an initial chart of the data according to those potential codes identified through familiarisation and created a further refined list of codes based on that. The Belgian team continued the familiarisation process by rereading the transcripts and creating a separate list of initial codes. We then brought those codes together and refined both lists into one.
4. Developing an analytical framework	Organising codes according to categories and trialling and redefining the organised coding framework until the coding framework reflects the whole data set.	The refined list produced from open coding was organised into a framework that grouped codes into categories collaboratively by the UK and Belgian teams. The framework was trialled independently by both teams on a selection of two transcripts, and then redefined to better suit the data based on the coders’ findings. That process was repeated three times until the framework was considered to reflect the dataset as a whole.
5. Applying the analytical framework	Using the finalised analytical framework to code the dataset.	The finalised framework was applied to the dataset; the Belgian team coded the Belgian transcripts, and the UK team coded the Italian transcripts. The Belgian team coded the transcripts in Dutch for ease of understanding but used the English framework. The Italian transcripts were coded in English.
6. Charting the data into a framework matrix	Inputting the coded data into a framework matrix that is organised by category and code for each participant. Coded data are inputted through a mixture of summarisation and direct quotations.	Data were input into an Excel file that was organised by participant, category and codes. Italian transcript data were input in English and Belgian transcript data were input in Dutch, and then translated to English to that the whole team could interpret the data.
7. Interpreting the data	Developing a set of themes through identifying the salient issues covered in the data.	Both teams examined the framework matrix independently to create initial lists of potential themes by identifying salient issues that were evident in the data. Themes were discussed and continually reappraised alongside the coded data until they were considered to accurately depict the meaning relayed by the dataset as a whole whilst remaining interpretive as opposed to descriptive.

**Coding Framework**

Each different highlighted colour represents a category. Codes in *italics* are codes that were added from familiarisation/open coding of the Italian data; the rest of the codes were created from the Belgian data.

**Table A2:** Coding framework.

**1. Cognitions associated with loneliness—the things that young people perceived that young people experiencing loneliness would think about**	
1a. Imagining interactions that they are missing out on	Fantasising about fun they could have if they had friends, and what they might be missing out on
1b. Considering how the future may look	Thinking about how the future might look for them, sometimes feeling that things might get better or feeling a lack of hope about their future
1c. Thinking negatively about themselves	Blaming themselves for how they feel, or for not being able to join in with peers and make friends. This is where the interviewee notes that young people may think of themselves as responsible for their loneliness
1d. Lack of trust in others	Finding it difficult to trust people enough to build connections
1e. Attempting to think positively	Trying to think of happy situations in order to lift their negative mood
1f. Thinking negatively about having less friends/confidants	Young people described how someone who is lonely may feel sad if they think that they have deficits in their relationships in comparison to their peers
1g. Negative thoughts and feelings directed at peers	Directing negative emotions such as anger and jealousy, towards peers
**2. Recognisable personal characteristics of someone who is experiencing loneliness—young people described personal attributes that they thought someone experiencing loneliness would have**	
2a. Emotions associated with loneliness	Emotions that young people use to describe the feelings of those experiencing loneliness
2b. Bottling things up/not expressing self	Describing how someone feeling lonely keeps their emotions to themselves
2c. Sensitive to negativity	Describing how someone feeling lonely may be more easily upset by things their peers say to them, or to fall outs and conflict than their peers who are not experiencing loneliness
**3. Recognisable social characteristics of someone who is experiencing loneliness—ways a young person experiencing loneliness is said to act in social situations**	
3a. Spending time away from others	Describing how someone feeling lonely keeps themselves separate from others such as by sitting alone at playtimes
3b. Has difficulty socialising	Describing how someone feeling lonely is seen to struggle connecting and building friendships with others. They might be seen as unusual to others and struggle with social skills, e.g., looking at people directly when talking to them, etc.
3c. Enjoys being alone	Describing how someone who is feeling lonely may enjoy being alone
3d. Searching for company	Describing how someone experiencing loneliness may spend time searching for friends
**4. Contexts and situations in which loneliness arises—what young people considered to be the things that triggered the experience of loneliness**	
4a. Being bullied or picked on at school	Stating that being the victim of bullying is a situation where someone might feel lonely
4b. Being excluded from socialising with peers	Being purposefully left out or avoided by peers stated as a reason why someone might feel lonely e.g., excluded from conversations, or games
4c. Having no, or little, family	Feelings of loneliness at home being attributed to not having family members, or having few family members to spend time with, play with, or to provide support at home
4d. Not having friends/being part of a group	Not having friends, or satisfying friendships as a reason for feeling lonely
*4e. Having no one to rely on*	*Young people thought that someone might feel lonely if they had no one to support them, or confide in.*
4f. Loneliness takes place at school	Loneliness was discussed as taking place at school
4g. Bereavement	Experiencing the passing of a family member, or someone close.
4h. A lack of opportunity to engage in positive activities	Feeling lonely due to a lack of opportunities to socialise, or do the things you enjoy e.g., sports teams, afterschool classes, etc.
4i. The influence of situational changes	Where loneliness is arises due to a change in circumstances such as parents going out and leaving them on their own, moving away, moving schools, friends moving away, etc.
4j. Conflict with those close to them	Young people said that loneliness may occur when someone is in conflict with their family, or others who are close to them.
**5. Relational problem solving behaviours to lessen loneliness—young people made suggestions about how people could support someone who is feeling lonely to feel less lonely**	
5a. Peers can approach first	Peers can help those who are feeling lonely by inviting them to talk or join in with games
*5b. Peers can give advice*	*Young people talked about how they would talk to a lonely peer about how to make friends*
5c. Teachers as problem solvers	Teachers can help lessen loneliness by giving advice on how to overcome issues with peers
5d. Teachers encourage socialisation	Teachers can help lessen loneliness by providing opportunities for young people to make friends such as through group work or encouraging the class to let the person who experiencing loneliness join in
5e. Parents provide comfort	Young people talk about how parents can lessen loneliness by providing hugs and a security
5f. Parents provide encouragement	Parents can encourage young people to socialise with peers
5g. Parents act as confidant	Young people talk about how they can confide in parents, or parents can encourage them to talk about their issues
5h. Peers can provide opportunities to engage in positive activities	Where peers organise an event for those experiencing loneliness to attend. This is where rather than inviting someone to join in with an already existing activity they organise something specifically for that person for example taking them for a coffee
**6. Personal coping strategies—** **young people made suggestions about strategies someone feeling lonely could engage in to lessen loneliness**	
6a. Reaching out to others	Young people experiencing loneliness can approach others to and ask to talk, join in with games, for support, etc.
*6b. Isolating themselves*	*Young people talked about how someone feeling lonely might isolate themselves further to avoid feeling saddened by difficulties making friends*
6c. Solitary activities	Young people experiencing loneliness can try to find activities to do alone that could make them feel better such as walking, cycling, playing video games, etc.
6d. Ways to express self without the need for a confidant	Young people experiencing loneliness can find ways to express themselves that do not require someone else such as writing in a diary, talking to a teddy, etc.
6e. Changing the way you act	Young people suggested a young person experiencing loneliness may benefit from changing the way they act in social situations, i.e., act in a more authentic way, try not to be too over the top, make more of an effort to engage with others
6f. Barriers to engaging in coping strategies	Young people recognised that some individuals experiencing loneliness may find it difficult to cope. For example, reaching out to others and building friendships with others can take a long time, and/or be overwhelming for some
**7. Differences between the two types of loneliness—young people recognised how the two types of loneliness; social and emotional, might impact young people**	
7a. Considering the type of loneliness that is worse to experience	Young people considered how individuals experiencing social, or emotional loneliness might feel, and if they thought it would be worse to be socially or emotionally lonely
7b. Reasons why a type of loneliness seemed worse	Young people talked about the reasons why they were more concerned with experiencing a certain type of loneliness
**8. Understanding of what determines experiences of loneliness—young people discussed what they thought loneliness was and the factors involved in experiences of loneliness that would be important to young people**	
8a. Gender differences in loneliness	Young people considered the ways boys and girls may experience loneliness
8b. Difficulty recognising that loneliness is different to being alone	Some young people found it difficult to differentiate between loneliness and being alone. Code used to identify where difficulties are identified
*8c. Loneliness comes from within*	*Young people talked about loneliness as a feeling that occurred even in the presence of others because it begins internally*
*8d. Loneliness develops as you age*	*Young people thought that loneliness could develop as a result of your emotions becoming more complex with age*

**Themes and Underpinning Codes**

Theme 1. **Loneliness Comes from Within**
  Underpinning codes: loneliness comes from within, enjoys being alone, emotions associated with loneliness.
Theme 2. **Loneliness as Missing Out**
  Subtheme: *A Lack of Desired Relationships*
    Underpinning codes: lack of trust in others, not having friends or being part of a group, imagining interactions they are missing out on, sensitive to negativity.
  Subtheme: *Difficulty Connecting with Others*
    Underpinning codes: has difficulty socialising, spending time away from others, bottling things up/not expressing self.
  Subtheme: *A Lack of Support*
    Underpinning codes: being excluded, conflict with those close to them, having little or no family, having no one to rely on.
Theme 3. **Loneliness and Thinking Negatively**
  Subtheme: *Thinking Negatively about One’s Self*
    Underpinning codes: thinking negatively about themselves, thinking negatively about having less friends or confidants, considering how the future may look.
  Subtheme: *Thinking Negatively about Others*
    Underpinning codes: negative thoughts and feelings directed at peers.
Theme 4. **Loneliness as a Transient Experience**
  Subtheme: *Loneliness According to Gender*
    Underpinning codes: gender differences.
  Subtheme: *Loneliness Changes as you Age*
    Underpinning codes: loneliness develops as you age.
  Subtheme: *Influence of Situational Changes*
    Underpinning codes: bereavement, the influence of situational change.
Theme 5. **Coping Strategies that Aim to Alleviate the Negative Emotional Impact of Loneliness**
  Underpinning codes: barriers to engaging in coping strategies, isolating themselves.
  Subtheme: *Expressing Thoughts to Self*
    Underpinning codes: ways to express self without confidant.
  Subtheme: *Solitary Activities to Distract*
    Underpinning codes: solitary activities.
  Subtheme: *Parents as a Source of Comfort*
    Underpinning codes: parents provide comfort, parents provide encouragement.
Theme 6. **Coping Strategies that Aid Social Reconnection**
  Subtheme: *Changing the Way You Act*
    Underpinning codes: changing the way you act, attempting to think positively, reaching out to others.
  Subtheme: *Peer Efforts towards Inclusion*
    Underpinning codes: peers approach first, peers provide opportunities to engage in positive activities, peers can give advice.
  Subtheme: *Teacher Encouragement of a Cohesive School Environment*
    Underpinning codes: teachers as problem solvers, teachers encourage socialisation.

**Secondary Analysis: Deductive Content Analysis**

To investigate patterns identified during the TFA, a deductive content analysis was conducted. The analysis aimed to explore the differences between coping strategies suggested by male versus female participants due to the finding by which participants suggested that males would be less likely to talk about their feelings of loneliness than females. The analysis also aimed to explore the type of loneliness, (i.e., loneliness due to having no one to play with, or share secrets with) that participants elected to be worse according to their age, and subsequently their reasons for making that choice. The analysis involved creating codes to group responses given by participants using the existing excel matrix of coded data to devise the codes. An alternative matrix was then created in order to record the counts of each new code for each participant. Frequencies were then calculated for each code. As regards gender, percentages were calculated out of the total number of participants for each gender, i.e., 12 males/12 females. As regards age, percentages were calculated according to the number of participants that were either age ranges 8–11 years or 12–14 years. For the analysis focusing on age Belgian participants only were included due to the range of ages being greater than that of the Italian participants. Coding was conducted by the first author and cross-checked by two other members of the research team; where disagreements occurred, newly agreed codes were devised and coding was reconducted.

**Table A3:** Coping strategies that alleviate negative feelings according to gender.

Codes	Male	Female
Confide in parents/family	41.67%(5)	33.33%(4)
Spend time alone	-	8.33%
Isolate themselves	16.67%(2)	8.33%(1)
Write down thoughts/feelings/secrets	25%(3)	33.33%(4)
Talk to yourself	16.67%(2)	8.33%(1)
Talk to a toy	-	8.33%(1)
Exercise	8.33%(1)	-
Distracting activities ^1^	16.67%(2)	16.67%(2)
Physical contact ^2^		

^1^ For example: reading, using technology, completing homework, hobbies. ^2^ For example: hugs.

**Table A4:** Coping strategies that aid social reconnection according to gender.

Codes	Male	Female
Peers inclusion	66.67%(8)	88.33%(10)
Teachers offer help/encourage socialisation	25%(3)	41.67%(5)
Change one’s attitude/behaviour	8.33%(1)	16.67%(2)
Peers/adults give advice	8.33%(1)	16.67%(2)
Peers listen/talk	50%(6)	16.67%(2)
Peers organise social events	8.33%(1)	8.33%(1)
Ask to join in with peers	25%(3)	50%(6)
Find/make new friends	41.67%(5)	58.33%(7)
Be more sociable	33.33%(4)	58.33%(7)
Focus on existing friends	16.67%(2)	-
Talk to/seek help from a professional	16.67%(2)	-
Reach out for help from parents/family	50%(6)	41.67%(5)
Parents can provide help/support	8.33%(1)	25%(3)
Express feelings ^1^	8.33%(1)	--

^1^ For example: creating art to show an adult how you feel.

**Table A5:** Type of loneliness according to age.

Codes	8–11 Years	12–14 Years
Social loneliness is worst ^1^	66.67%(4)	33.33%(2)
Emotional loneliness is worst ^2^	16.67%(1)	66.67%(4)
Both equal/same	16.67%(1)	-

^1^ This refers to feeling lonely because you do not have anyone to play with. ^2^ This refers to feeling lonely because you do not have anyone to talk to or share secrets with.

**Table A6:** Reasons for making choice of loneliness type.

Codes	8–11 Years	12–14 Years
Prefer having people to play with over sharing secrets	66.67%(4)	16.67%(1)
Importance placed on talking	-	66.67%(4)
Do not like sharing secrets	16.67%(1)	-
Playing with others is harder than talking	-	16.67%(1)
